# Tropomyosin Isoform Diversity in the Cynomolgus Monkey Heart and Skeletal Muscles Compared to Human Tissues

**DOI:** 10.1155/2023/1303500

**Published:** 2023-01-24

**Authors:** Dipak K. Dube, Syamalima Dube, Lynn Abbott, Omar Elsekaily, Samender S. Randhawa, Jean M. Sanger, Joseph W. Sanger, Bernard J. Poiesz

**Affiliations:** ^1^Department of Medicine, SUNY Upstate Medical University, 750 East Adams Street, Syracuse, NY 13210, USA; ^2^Department of Cell and Developmental Biology, SUNY Upstate Medical University, 750 East Adams Street, Syracuse, NY 13210, USA

## Abstract

Old world monkeys separated from the great apes, including the ancestor of humans, about 25 million years ago, but most of the genes in humans and various nonhuman primates are quite similar even though their anatomical appearances are quite different. Like other mammals, primates have four tropomyosin genes (TPM1, TPM2, TPM3, and TPM4) each of which generates a multitude of TPM isoforms via alternative splicing. Only TPM1 produces two sarcomeric isoforms (TPM1*α* and TPM1*κ*), and TPM2, TPM3, and TPM4 each generate one sarcomeric isoform. We have cloned and sequenced TPM1*α*, TPM1*κ*, TPM2*α*, TPM3*α*, and TPM4*α* with RNA from cynomolgus (Cyn) monkey hearts and skeletal muscle. We believe this is the first report of directly cloning and sequencing of these monkey transcripts. In the Cyn monkey heart, the rank order of TPM isoform expression is TPM1*α* > TPM2*α* > TPM1*κ* > TPM3*α* > TPM4*α*. In the Cyn monkey skeletal muscle, the rank order of expression is TPM1*α* > TPM2*α* > TPM3*α* > TPM1*κ* > TPM4*α*. The major differences in the human heart are the increased expression of TPM1*κ*, although TPM1*α* is still the dominant transcript. In the Cyn monkey heart, the only sarcomeric TPM isoform at the protein level is TPM1*α*. This is in contrast to human hearts where TPM1*α* is the major sarcomeric isoform but a lower quantity of TPM1*κ*, TPM2*α*, and TPM3*α* is also detected at the protein level. These differences of tropomyosin and/or other cardiac protein expression in human and Cyn monkey hearts may reflect the differences in physiological activities in daily life.

## 1. Introduction

Nonhuman primates play a critical role in various human disease research. Due to a high level of homology with human genes, *Macaca fascicularis*, the cynomolgus (Cyn) monkey, is one of the most widely used nonhuman primate models in biomedical research. They have been widely used for modeling human disorders such as Parkinson's disease [1]. Recently, Seita et al. [2] have generated transgenic Cyn monkeys that over express the Amyloid-*β* Precursor Protein gene for use in Alzheimer research.

Vertebrate cardiac muscle, the cross-striated muscle of the heart, helps contract the heart, which is necessary for pumping blood towards the lungs and throughout the body. A cooperative interaction between thick and thin filaments in cardiac muscles generates the muscle contraction [3, 4]. It is well established that tropomyosin (TPM), a component of thin filament, interacts with the actin and troponin complex to control the contractile activity [5–9]. Different isoforms of myofibrillar proteins, for example, TPM, may variably regulate muscle contraction. In order to understand the role of any myofibrillar protein in muscle contraction in any organism, it is essential to know the expression pattern of various isoforms of each of the myofibrillar proteins.

Alternate splicing can produce a vast number of spliced transcripts of all mammalian TPM isoforms [5–7]. However, we have very little knowledge about the range of splicing of monkey TPM transcripts. As mentioned earlier, the monkey is one of the most useful animal models to study various human diseases including heart diseases. Humans share over 90% of their DNA with other primates, for example, chimpanzees and monkeys (https://www.sciencedaily.com/releases/2012/11/121106201124.html). Many phenotypic differences between humans and nonhuman primates are probably due to more changes in gene regulation than differences between the genes themselves [10]. Our current goal is to explore the isoform diversity of various TPM genes in striated muscles of Cyn monkeys. We have amplified, cloned, and sequenced cDNAs of various sarcomeric isoforms. Nucleotide sequence analyses gave us insight into all different TPM isoforms.

The expression patterns of each of the transcripts in the monkey heart and skeletal muscle were determined by qRT-PCR. These results were compared to those obtained from similar human tissues. Using two-dimensional western blotting with monkey heart lysate and CH1 monoclonal antibody specific against vertebrate striated muscle TPM isoforms [11, 12], we separated various sarcomeric TPM isoforms and subsequently performed mass spectra analyses to determine the expression pattern of TPM isoforms in monkey heart.

## 2. Materials and Methods

Total RNAs from heart and skeletal muscle of adult Cyn monkey were procured from BioChain (Newark, CA). The lot numbers of heart and skeletal muscle extracts are B409007 and B308110, respectively. The animals were adult and healthy. The heart and skeletal muscle samples were not necessarily from the same animal. Cyn monkey heart extracts for 2D western blot analyses were procured from BioChain (Lot# A705219) and Zyagen, San Diego, CA (Cat# KT-801).

Normal adult human heart RNA (Lot # B712083) was procured from BioChain (Newark, CA). Normal adult human skeletal muscle RNA was obtained from Biochain (Cat # R1234171-50) and Stratagene (Cat # 540024-41).

### 2.1. RT-PCR for Amplification of TPM1*α*, TPM1*κ*, TPM2*α*, TPM3*α*, and TPM4*α*

cDNAs were made from various RNAs using oligo dT (unless mentioned otherwise) using our published protocols [8, 13–15]. Subsequent PCR-amplification of gene and/or isoform specific isoforms were carried out with isoform specific primer-pairs as given in Table 1. The PCR amplified DNA were separated by agarose gel electrophoresis and subsequently stained with ethidium bromide as stated before [15]. Various ethidium bromide stained DNA bands were excised from agarose gel and DNA was extracted using the MiniElute Gel extraction kit (Qiagen, Velencia, CA). The extracted DNA was sent for sequencing. Also, a portion of each gel extracted DNA was used for cloning into T/A cloning vectors (Life Technologies, Carlsbad, CA) following our published protocol [13]. The DNA from the positive clones were extracted with Qiagen mini-prep kit (Valencia, CA). Each of the isolated DNA in T/A cloning vector was sequenced from both directions (Cornell University Life Science Core Laboratories center, Ithaca, NY).

### 2.2. Real-Time Quantitative RT-PCR

In order to quantify transcript level in a given tissue one can determine both relative quantification and absolute quantification. Relative quantification is used to relate the amount of the transcripts of the gene of interest in equivalent amounts of different samples. However, the absolute quantification provides the copy number of the target gene present in the sample. Relative quantification of qRT-PCR data was performed using the ΔCT and ΔΔCT methods [16–19].

The reaction mixture contained 12.5 *μ*l of the SYBR green supermix, 1 *μ*l of both positive and negative 10 mM primer, 9.5 ml DEPC-treated H_2_O, 1 ml of cDNA for the unknowns, 1 *μ*l of DNA from the dilution series of each TPM TA clones for the standards, or 1 *μ*l of H_2_O for the primer control. To verify the specificity of the primer pair, PCR products were run on an agarose gel after real-time analysis. For qRT-PCR of TPM1*α*, TPM2*α*, TPM3*α*, and TPM4*α*, cDNA for each isoform was made with the corresponding gene and isoform-specific oligonucleotide designed from the exon 9 A/B of the respective TPM genes. The strategy of qRT-PCR was used for maintaining the specificity (or avoiding the cross amplification) of the highly conserved genes such as TPMs. The nucleotide sequences for isoform-specific oligonucleotides used for making cDNA are given in Table 1.

The absolute copy number was determined by standard curve method as described previously [14, 15, 20].

### 2.3. 2D Western Blot and Mass Spectrometry (LC-MS/MS)

Extracts of normal adult hearts of Cyn were procured from Zyagen (San Diego, CA, USA) and BIoChain Institute, Inc., CA. 2D Western blot analyses was carried out by Kendrick Labs using their published protocol [21, 22] as described previously [14, 23]. A superimposition of X-ray film and the Coomassie stained protein gel exhibited four spots for each sample (Supplementary Figures 4 and 6). Mass spectra analyses were performed with excised, washed, and trypsinized proteins from each gel spot as described before [24–26].

### 2.4. Data Processing and Protein Identification

ProteinLynx Global Server (PLGS, version 2.4) was used for processing the raw data and protein identification (https://www.matrixscience.com/, Matrix Science, London, UK) [14, 23].

## 3. Results

### 3.1. Cloning and Sequencing of Two Sarcomeric Isoforms of the TPM1 Gene

**Figure 1:**
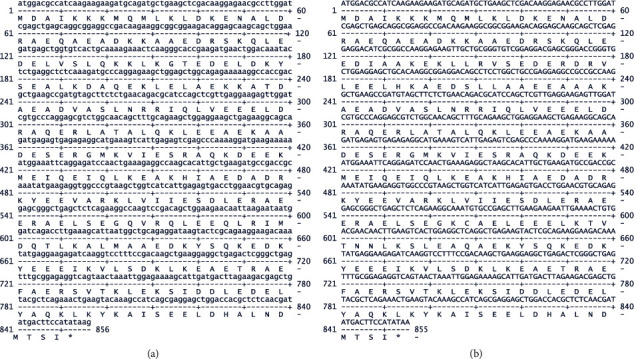
The nucleotide sequence and derived amino acid sequence of cDNA coding for Cyn heart (a) TPM1*α* and (b) TPM1*κ*.

It is well established that the mammalian TPM1 gene generates two sarcomeric isoforms known as TPM1*α* (or TPM1.1) and TPM1*κ* (or TPM1.2) [8, 11]. Two additional high molecular weight isoforms, TPM1*μ* and TPM1*ξ* have been identified in human breast cell lines but not in human cardiac tissue [15]. Although the predicted nucleotide sequences of TPM1*α* from various monkeys are known, to the best of our knowledge, no one has reported TPM1*α* and TPM1*κ* actual nucleotide sequences from Cyn monkey in the literature. Hence, we decided to clone and sequence the cDNAs of TPM1*α* and TPM1*κ* from Cyn monkey striated muscles. Because TPM1 sequences of Cyn monkey are not available in the databases, we designed a number of primer-pairs for PCR amplification from the predicted TPM1*α* sequences of *Macaca mulatta* (MM) available in the database (variant X5 (XM_001103963)). We chose MM because these are also old-world monkeys such as Cyn. cDNAs made from the RNA of Cyn monkey heart and skeletal muscle with oligo dT were used for PCR amplification. First PCR amplification was performed with TPM1 exon 1A (+) and TPM1 exon 9B (−) primer-pair (Table 1), which would amplify both TPM1*α* and TPM1*κ*. The amplification strategy of TPM1*α*, TPM1*κ*, TPM1*μ*, with RNA from Cyn heart and skeletal muscle are described in Supplementary Figure 2 and Supplementary Table 1. As stated in the supplementary section, TPM1*κ* was amplified and divided in two parts using primer pairs P4(+)/P3(−) and P(1)/P6(−) as stated in Supplementary Table 1. The nucleotide sequences of TPM1*α* and TPM1*κ* are depicted in Figures 1(a) and 1(b), respectively. Also, we have compared the nucleotide sequences of Cyn TPM1*α* and TPM1*κ* with human TPM1*α* (NM_001018005.1) and Human TPM1*κ* (accession number), respectively. The comparative nucleotides CynTPM1*α* VS. Human TPM1*α* and CynTPM1*κ* VS. Human TPM1*κ* are shown in Supplementary Figures 3A and 3B, respectively.

### 3.2. Cloning and Sequencing of TPM2*α*

cDNAs were made with RNA from the monkey heart and skeletal muscle with oligo dT as described under Materials and Methods section. Initial PCR amplification was performed with TPM2 exon 1A(+)/TPM2 Exon 9A2(−) primer pairs. The PCR-amplified DNAs were separated in an agarose gel, and DNA was extracted from the topmost gel band for direct sequencing and also cloning into T/A cloning vector [14].

Although there are ∼2.6% differences in nucleotide sequences between human and Cyn sequences of TPM2a (Supplementary Figure 3C), the deduced amino acid sequences are identical (accession # NM_003289.4)

### 3.3. Cloning and Sequencing of TPM3*α*

**Figure 2:**
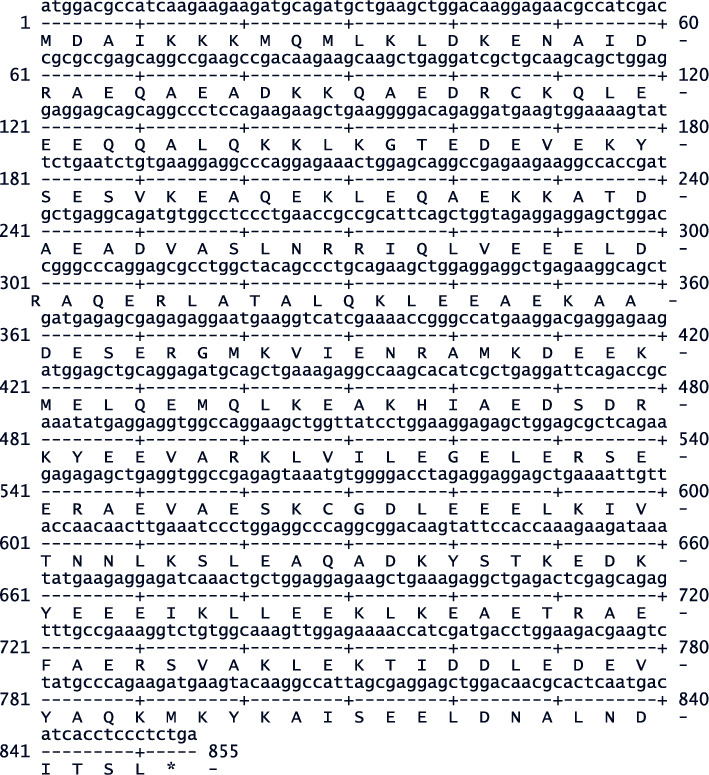
Nucleotide sequence of Cyn heart TPM2a cDNA and its deduced amino acid sequence.

**Figure 3:**
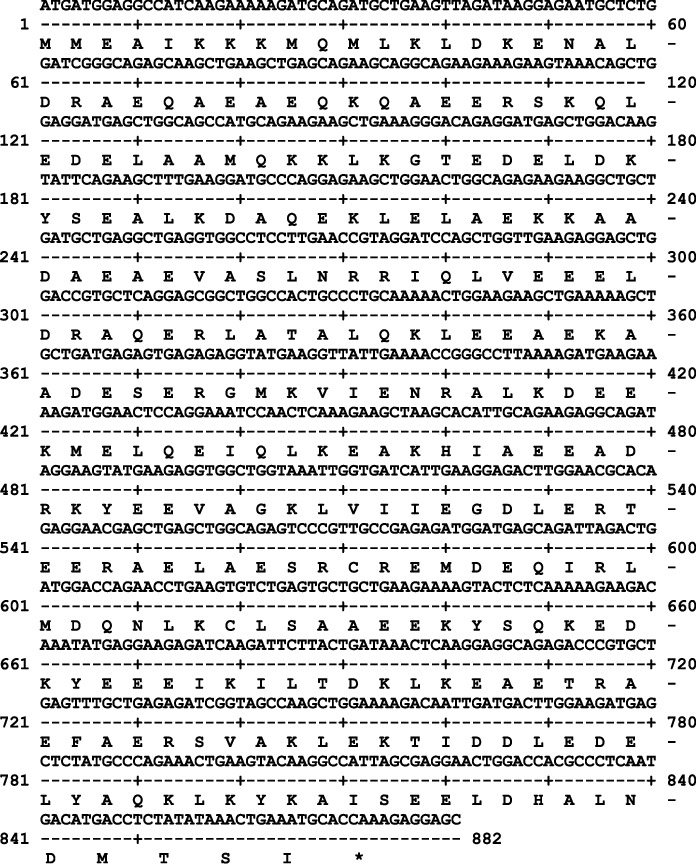
The nucleotide sequence of cDNA and deduced amino acid sequence of Cyn heart TPM3*α*.

Amplification of TPM3a has been described in the supplementary section. The nucleotides as well as deduced amino acid sequences are shown in Figures 2 and 3. It is to be noted that although amino acid sequence of Cyn and human TPM3a are identical but there the nucleotide sequences are 98.481% similar. The best fit of the two nucleotide sequences are shown in Supplementary Figure 3D.

### 3.4. Cloning and Sequencing of TPM4*α*

**Figure 4:**
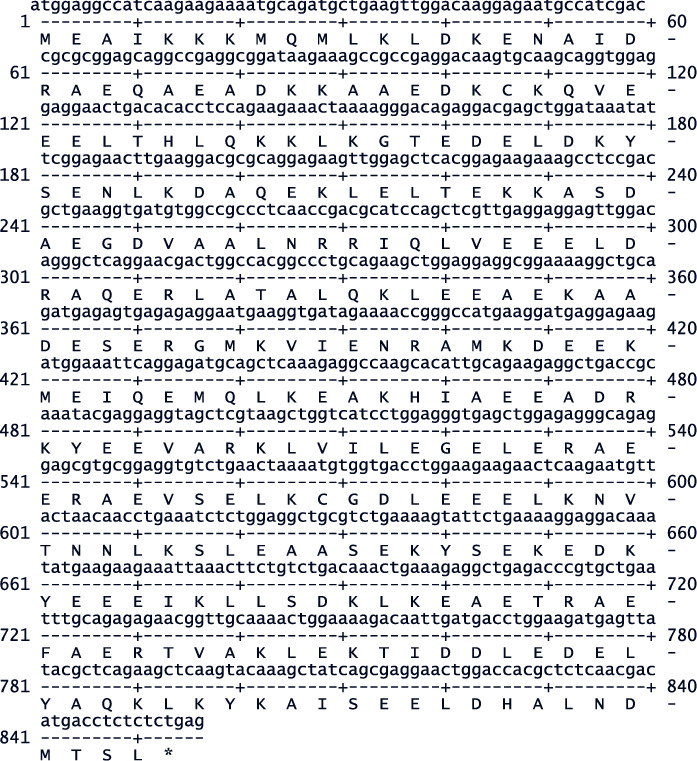
The nucleotide sequence and derived amino acid sequence of cDNA for Cyn heart TPM4*α*.

cDNAs were prepared for monkey TPM4*α* from RNA with oligo dT as stated above for TPM2*α*. The primer-pair used for initial amplification was TPM4 Exon 1A(+)/TPM4 Exon 9A(−) (nucleotide sequences are depicted in Table 1). The second primer-pair used for screening clones is TPM4 qRT(+)/TPM4 Exon 9A(−). The nucleotide sequences of monkey TPM4*α* (Figure 4) is ∼98.13% identical with the human TPM4*α* sequences whereas the amino acid sequences are identical [27]. The best fit results of CynTPM4*α* and Human TPM4*α* nucleotide sequences are depicted in Supplementary Figure 3E. The nucleotide sequences of Cyn TPM4*α* and human TPM4*α* are 98.13% similar.

### 3.5. Quantitation of Transcripts of Various High Molecular Weight TPM Isoforms in the Monkey Heart and Skeletal Muscle

Quantification of the transcript level of a specific isoform of any gene in a given tissue can be achieved by both relative quantification and absolute quantification. Relative quantification is used to relate the amount of the transcripts of the gene of interest in equivalent amounts of different samples. However, the absolute quantification provides the copy number of the target gene present in the sample. Again, the relative expression can be determined by two methods, by ΔCt and by 2^−ΔΔCt^. In this study, we have evaluated relative expression using both methods and we have assessed the absolute copy number of various TPM isoforms in Cyn monkey heart and skeletal muscle. Also, we have performed comparative analyses for the expression of TPM isoforms between human and Cyn monkey.

### 3.6. Relative Expression of TPM1 Isoforms in the Heart and Skeletal Muscle

The main differences between TPM1*α* and TPM1*κ* is in exon 2, whereas other exons including UTRs are the same. As the nucleotide sequences of the coding regions of various TPM isoforms are very similar, we made cDNA with gene and isoform specific oligonucleotides from the 3′-UTR (exon 9b) of TPM1*α* and TPM1*κ*, which precludes the amplification of other tropomyosin gene products.

**Figure 5:**
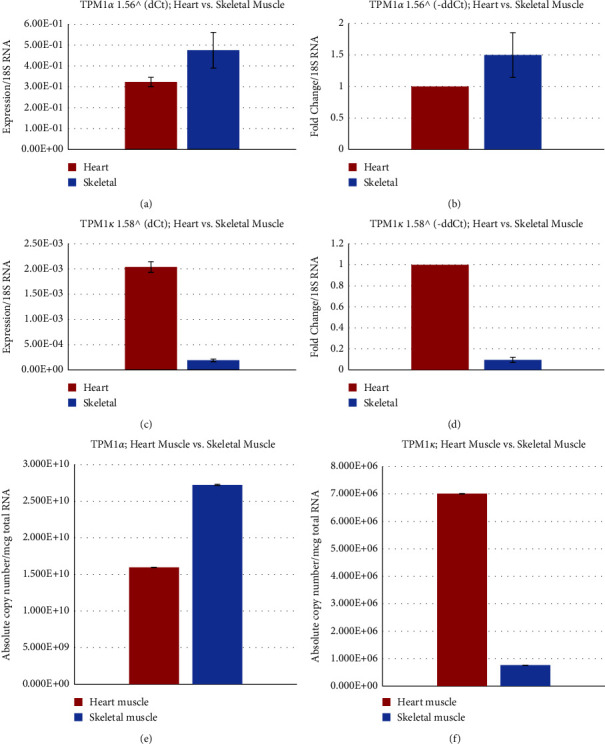
Relative and absolute expression of TPM1*α* and TPM1*κ* in the Cyn heart and skeletal muscle. (a) Relative expression of TPM1*α* using the dCt method. (b) Fold change of TPM1*α* using the ddCt method. (c) Relative expression of TPM1*κ* using the dCt method. (d) Fold change of TPM1*κ* using the ddCt method. (e) Estimation of the absolute copy number of TPM1*α*. (f) Estimation of the absolute copy number of TPM1*κ*.

Figure 5(a) depicts the relative expression of TPM1*α* in Cyn monkey heart and skeletal muscle using ΔCt method that shows the higher expression level of TPM1*α* in skeletal muscle. Also, a higher fold TPM1*α* expression level was recorded in skeletal muscle when we used the ΔCt method as shown in Figure 5(b).  Figure 5(e) and Table 2 show that the absolute copy number of TPM1*α* transcripts per mcg of total cellular RNA is ∼1.7 fold higher in skeletal muscle. The copy number results are in agreement with the relative expression results as presented in Figures 5(a) and 5(b). On the contrary, TPM1*κ* expression in Cyn monkey heart is significantly higher than in skeletal muscle as determined by both ΔCt (Figure 5(c)) and ΔΔCt method (Figure 5(d)). The higher TPM1*κ* expression in Cyn monkey heart is supported by the expression results determined by absolute copy number (Figure 5(f) and Table 2). However, compared to TPM1*κ*, the expression of TPM1*α* is 2.3 × 10^3^ and 3.56 × 10^4^ fold higher in the heart and skeletal muscle, respectively (comparison of results in Figures 5(b) and 5(e) and Table 2).

**Figure 6:**
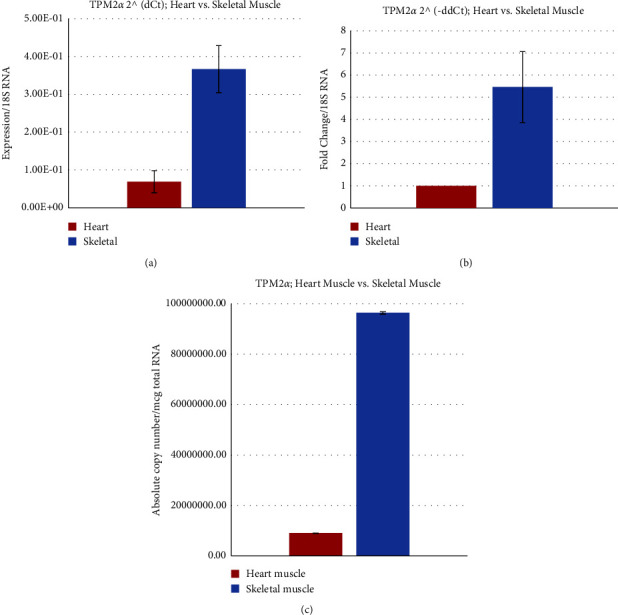
Relative and absolute expression of TPM2*α* in Cyn striated muscles. (a) Relative expression of TPM2*α* using the dCt method in the Cyn heart and skeletal muscle. (b) Fold change of TPM2*α* in the Cyn heart and skeletal muscle using the ddCt method. (c) Determination of the absolute copy number of TPM2a in the Cyn heart and skeletal muscle.

The relative expression data as determined by ΔCt (Figure 6(a)) and ΔΔCt (Figure 6(b)) show that TPM2*α* transcript level is much higher in skeletal muscle. The higher expression level of TPM2*α* in Cyn monkey skeletal muscle is also corroborated by the determination of absolute copy number. It is ∼10.5 fold higher in skeletal muscle compared to cardiac muscle (Figure 6(c) and Table 2).

In humans, the expression of TPM2*α* is ∼3.9 × 10^2^ higher in skeletal muscle compared to cardiac tissue. TPM2*α* level in human and Cyn monkey hearts is comparable but it is ∼5 fold higher in skeletal muscle in humans compared to Cyn monkey (Table 2).

**Figure 7:**
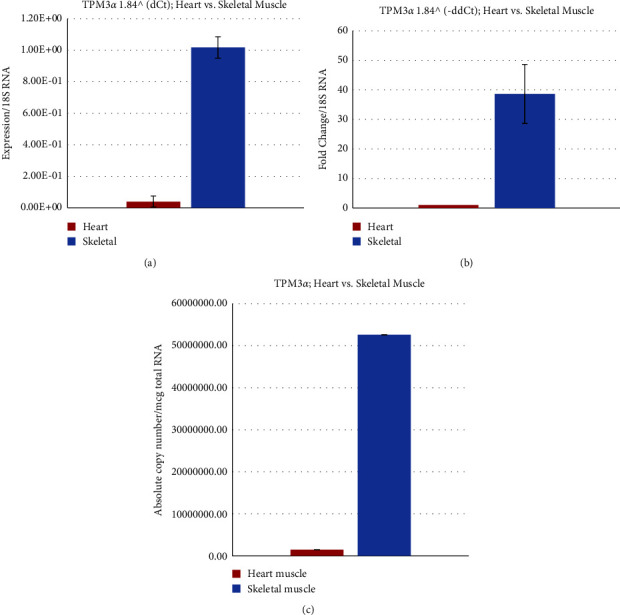
Relative and absolute expression of TPM3*α* in the Cyn heart and skeletal muscle. (a) Relative expression of TPM3*α* using the dCt method. (b) Fold change of TPM3*α* using the ddCt method. (c) Estimation of the absolute copy number of TPM3*α* in the Cyn heart and skeletal muscle.

The relative expression of TPM3*α* is significantly higher in Cyn monkey skeletal muscle compared to cardiac muscle (Figures 7(a) and 7(b)). Again, absolute copy number data as presented in Figure 7(c) and Table 2 point out a ∼36.5 fold higher expression of TPM3*α* in skeletal muscle.

The expression of TPM3*α* is 13 fold higher in human skeletal muscle compared to human cardiac muscle. The expression of TPM3*α* in human cardiac muscle is about 11 fold higher than Cyn cardiac muscle. The expression of TPM3*α* in human skeletal muscle is about 4 fold higher than in Cyn skeletal muscle.

**Figure 8:**
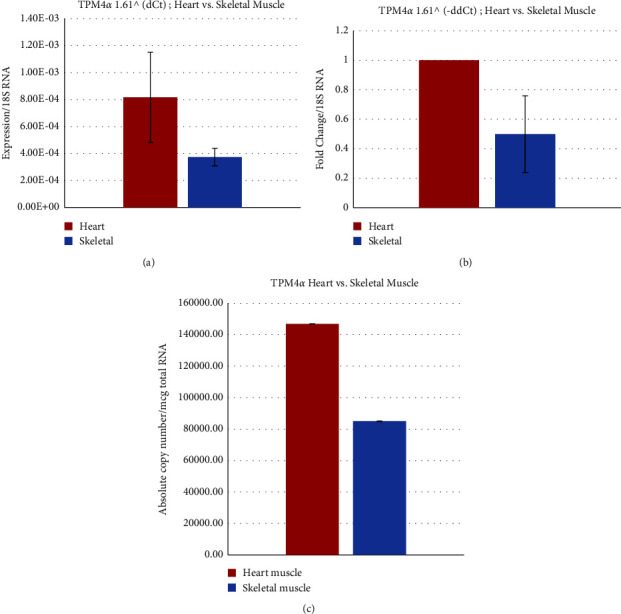
Relative and absolute expression of TPM4*α* in Cyn striated muscles. (a) Relative expression of TPM4*α* using the dCt method in the Cyn heart and skeletal muscle. (b) Fold change of TPM4*α* in the Cyn heart and skeletal muscle using the ddCt method. (c) Determination of the absolute copy number of TPM4a in the Cyn heart and skeletal muscle.

The relative expression (Figures 8(a) and 8(b)) as well as absolute expression of TPM4*α* (Figure 8(c) and Table 2) are higher (1.7 times) in monkey cardiac muscles compared to the skeletal muscle. The expression of TPM4*α* is about the same in human heart vs. human skeletal muscle. The expression of TPM4*α* in human cardiac muscle is 19 fold less compared to the Cyn cardiac muscle. The expression of TPM4*α* in human skeletal muscle were about 8 fold less than in Cyn skeletal muscle.

Table 2 shows that TPM1*α* transcripts are 1.13 fold higher in the human heart compared to human skeletal muscle, whereas TPM1*κ* is 67.7 fold higher in the heart. The expression of TPM1*α* is 1.24 × 10^2^ and 3.2 × 10^4^ fold higher than TPM1*κ* in the human heart and skeletal muscles, respectively. The expression of TPM1*α* is very similar in the human heart compared to the Cyn heart, whereas the expression of TPM1*κ* is 22.4 fold higher in the human heart. Likewise, the expression of TPM1*α* in the monkey and human hearts is very similar, while the expression of TPM1*κ* is about 3 fold greater in the human skeletal muscle compared to Cyn skeletal muscle.

Determination of absolute copy number helps us to appraise the comparative expression of various TPM isoforms in Cyn hearts where TPM1*α* > TPM1*κ* > TPM2*α* > TPM3*α* > TPM4*α*. On the contrary, Cyn skeletal muscles express TPM1*α* > TPM2*α* > TPM3*α* > TPM1*κ* > TPM4*α*. In human hearts, TPM1*α* > TPM1*κ* > TPM3*α* > TPM2*α* > TPM4*α*. In human skeletal muscle, TPM1*α* > TPM2*α* > TPM3*α* > TPM1*κ* > TPM4*α*.

### 3.7. 2D Western Blot Analyses of Cyn Monkey Cardiac Muscle Protein Extract with CH1 Monoclonal Antibody Followed by LC-MS/MS Analysis

**Figure 9:**
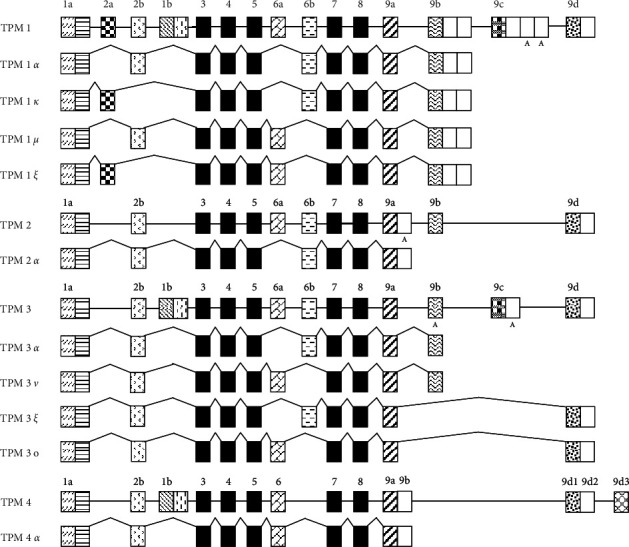
Alternative splicing patterns of TPM genes in human/nonhuman primates that generate high molecular weight isoforms. Exon compositions of TPM1, TPM2, TPM3, and TPM4 are derived from various published documents, the recently submitted data, and the predicted sequences available in Gen Bank. Exons are shown in boxes.

We carried out 2D western blot analyses with extracts from two different monkey hearts with CH1 monoclonal antibody specific for sarcomeric TPM proteins. Peptides were extracted from CH1 positive spots for subsequent LC-MS/MS analyses. Mass spectra data and analyses are presented in Supplementary Figures 5 and 7 in the supplementary section. The results depicted in Table 3 show that 80% of the identified TPM peptides are specific for TPM1 and we failed to detect any TPM2, TPM3, or TPM4 specific peptide. It is not illogical if one concludes the absence of TPM2, TPM3, and TPM3 protein in all four spots. In other words, only TPM1 protein is present in this heart extract. Next question is which TPM1 isoform is expressed. It is to be noted that 15 TPM1 specific peptides belong to TPM1*α* and/or TPM1*μ*. The difference between TPM1*α* and TPM1*μ* is in exon 6. TPM1*α* has exon 6B whereas TPM1*μ* contains exon 6A (Figure 9). Although we first detected the expression of TPM1*μ* transcript in human breast cancer cells [13], we are yet to detect the expression of TPM1*μ* protein in human striated muscles. As we have not identified any exon 6A specific peptide in either of the protein extracts (Tables 3 and 4), we conclude that the only sarcomeric TPM1 protein in the monkey heart is TPM1*α*. Our results are in good agreement with the results of Hu et al. [28], who also found the expression of only one high molecular weight sarcomeric TPM1 protein in the heart of Rhesus monkey, which is also an old-world monkey such as Cyn.

## 4. Discussion

Cloning, sequence analyses, and subsequent protein expression patterns of sarcomeric isoforms of TPM1, TPM2, TPM3, and TPM4 genes support the conclusion made by several well-known scientists that most human-monkey (chimp) differences are due to gene regulation and not genes. Nucleotide as well as deduced amino acid sequence analyses show that there is not much difference between human and monkey regarding TPM isoforms. The levels of expression of transcripts from various TPM isoforms in heart and skeletal muscles are also comparable between human and monkey. However, the expression level of TPM1*κ* transcripts in monkey heart is higher compared to other vertebrate hearts with the exception of humans (11 and the present study). In the monkey heart, the expression is TPM1*α* > TPM1*κ* > TPM2*α* > TPM4*α* > TPM3*α*, whereas the expression in monkey skeletal muscle is TPM1*α* > TPM2*α* > TPM3*α* > TPM1*κ*> TPM4*α* (Figures 5–8).

Although the expression pattern of transcripts of various sarcomeric TPMs in Cyn vs. human muscles are similar, the expression pattern of the corresponding proteins are strikingly different. We have detected the presence of TPM1*α* protein in Cyn hearts only (Tables 3 and 4). Currently, we do not have any explanation for the lack of other sarcomeric TPM expression in Cyn heart in spite of the presence of detectable quantities of TPM1*κ*, TPM2*α*, TPM3*α*, and TPM4*α* transcripts other than translational control. Our results are in good agreement with those of Hu et al. [28] who also detected only TPM1*α* protein isoform in cardiac tissue from three rhesus macaques, another old-world monkey species such as Cyn.

These results in Cyn are in contrast with humans, while TPM1*α* is the major sarcomeric TPM isoform in the heart; a lower quantity of TPM1*κ* expression has also been detected by us and several other laboratories as well [8, 11, 15, 29, 30]. Also, a lower quantity of TPM2*α* [11, 29, 30] and TPM3*α* protein [12, 30] has been detected in human hearts.

The primate lineage is thought to be ∼60 million years old [31]. Old-world primates diverged from a common ancestor to new-world primates ∼31 million years ago. The chimpanzees and humans diverged from other great apes ∼6-7 million years ago [32]. The genus, homo, evolved ∼2 million years ago and scientists have shown how drastically evolution has changed various organs such as brain and heart [33]. Shave et al. [34] reported extensive studies comparing the shape of hearts and various activities of chimpanzees, gorillas, and humans. Although gorillas and chimpanzees spend a lot of time sleeping or being relatively inactive, they can be extremely active in short bursts of resistance physical activity (RPA) such as climbing trees and fighting among themselves. These types of intense activities may create a pressure stress on the cardiovascular system. Monkeys may also follow similar pattern of activities. On the contrary, humans during their early development spent a lot of time for hunting, gathering, and later farming for their survival. In other words, humans for their survival depend on lifelong moderate-intensity endurance physical activity (EPA), which creates a cardiovascular volume stress. When left ventricular (LV) structure and function were compared, Shave et al. [34] showed that human LV possesses features that augment cardiac output, thereby enabling EPA. In addition, human LV also demonstrate phenotypic plasticity as well as variability of various physical activities. These findings clearly suggest functional differences between human and monkey hearts. Hence, it is arguably logical to detect differences in tropomyosin isoforms and other cardiac specific proteins expression in human and nonhuman primate hearts. An unaddressed question is why mRNAs for different sarcomeric TPM isoforms are made if the corresponding proteins are not required for various cardiac activities. Is it for emergency use if and when they are needed? The absence of various TPM protein in monkey hearts, however, can be explained by translational control of the corresponding transcripts in monkey hearts.

## Figures and Tables

**Table 1:** Nucleotide sequences of the oligonucleotides used for primers and/or probes.

TPM1	Type of amplification		Nucleotide sequence
Primer	PCR/RT-PCR	qRT-PCR	
TPM1 exon 1A (+)	X	—	5′-ATGGACGCCATCAAGAAGAA-3′ (A1+)
TPM1 exon 9A (−)	X	—	5′-AAGTCATATCGTTGAGAGCG-3′ (A2−)
TPM1 exon 9B (−)	X		5′-TAAGAGAGAGAACCAGGGTC-3′ (A3−)
TPM1 exon 2A (−)	X		5′-TGTCCTCCGCCTTGTGCAG-3′ (A6−)
TPM1 exon 2A (+)	X	X	5′-GAAGTTGCTGCGGGTGTCGG-3′ (A4+)
TPM1 exon 2B (+)	X	X	5′-TGGAAGATGAGCTGGTGTTAC-3′ (A5+)
TPM1 exon 3-4 (−)	X	X	5′-TCAATGACTTTCATGCCTCT-3′ (A7−)
TPM1 exon 6A (+)	X		5′-GAAAGCATTAATGGCTGCAGAG-3′ (A8+)
TPM1 exon 6B (+)	X		5′-TGAAGTCACTGGAGGCTCAGG-3′ (A9+)
TPM2 exon 1A (+)	X		5′-ATGGACGCCATCAAGAAGAA-3′
TPM2 exon 3 (+)	X		5′-ATTCAGCTGGTTGAGGAGGAGCTGG-3′
TPM2 exon 6B (+)	X		5′-TAAATGTGGGGACCTAGAGGAGGAG-3′
TPM2 RT-1 (+)		X	5′-CGGACAAGTATTCCACC-3′
TPM2 exon 9A (−)	X	X	5′-CTTGTACTTCATCTTCTGGGCATAG-3′
TPM2 exon 9A2 (−)	X		5′-AGGGAGGTGATGTCATTGA-3′
TPM3			
TPM3qRT (+)		X	5′-CTTGGAGCGCACAGAGGAAC-3′ (B1+)
TPM3qRT (−)		X	5′-GATCCAGAACAGAGCAGAAAC-3′ (B2−)
TPM3 exon 1A (+)	X		5′-ATGATGGAGGCCATCAAGAA-3′ (B3+)
TPM3 exon 6B (−)	X		5′-TCCAGCTCAGAAGACTTA-3′ (B4−)
TPM3 exon 6A (−)	X		5′-TTCTGGTCCATCAGTCTA-3′ (B5−)
TPM3 exon 9A (−)	X		5′-GCTAATGGCCTTGTACTTCAG-3′ (B6−)
TPM3 exon 9B (−)	X		5′-AATGGAATCCAGAGCGAGAGT-3′ (B7−)
TPM4			
TPM4qRT (+)		X	5′-CAGCCATGGAGGCCATCAAGA-3′
TPM4qRT (−)		X	5′-GCGTCGGTTGAGGGCGGCCAC-3′
TPM4 exon 9A (−)	X		5′-CTGCCTCTCAGAGAGAGGTC-3′
TPM4 exon 1A (+)	X		5′-ATGGAGGCCATCAAGAAG-3′
TPM4 exon 7 (+)	X		5′-ATTAAACTTCTGTCTGACAA-3′
18S rRNA			
Forward (+)		X	5′-TGCTGCAGTTAAAAAGCTCGTA-3′
Reverse (−)		X	5′-ACCAACAAAATAGAACCGCGGT-3′

**Table 2:** Expression of transcripts of various sarcomeric TPMs in heart and skeletal muscles in human and nonhuman primate.

Isoform	^*∗*^ Human heart	Monkey heart	Human skeletal muscles	Monkey skeletal muscles
TPM1*α*	1.95 × 10^^10^ ± 1.94 × 10^^7^	1.60 × 10^^10^ ± 6.04 × 10^^6^	1.73 × 10^^10^ ± 4.70 × 10^^6^	2.72 × 10^^10^ ± 6.80 × 10^^7^
TPM1*κ*	1.57 × 10^8 ± 5.30 × 10^^5^	7.01 × 10^^6^ ± 8.90 × 10^^3^	2.32 × 10^^6^ ± 2.80 × 10^^3^	7.65 × 10^^5^ ± 6.98 × 10^^2^
TPM2*α*	1.40 × 10^7^ ± 9.40 × 10^^1^	9.00 × 10^^6^ ± 6.20 × 10^^4^	5.50 × 10^^8^ ± 4.90 × 10	9.60 × 10^^7^ ± 4.50 × 10^^5^
TPM3*α*	1.60 × 10^7^ ± 3.20 × 10^3^	1.40 × 10^^6^ ± 2.70 × 10^^3^	2.10 × 10^8^ ± 1.90 × 10^^4^	5.26 × 10^^7^ ± 2.90 × 10^^4^
TPM4*α*	7.70 × 10^^3^ ± 4.70 × 10	1.46 × 10^^5^ ± 1.50 × 10^^2^	1.03 × 10^^4^ ± 1.70 × 10	8.50 × 10^^4^ ± 4.00 × 10

cDNA from human and Cyn RNA were made with the same oligonucleotide(s) and amplified with the same primer pair.

**Table 3:** Tropomyosin peptides identified in adult monkey heart #1.

Spot	TPM1			TPM2			TPM3			TPM4		
	Total	Unique	Isoform	Total	Unique	Isoform	Total	Unique	Isoform	Total	Unique	Isoform
Spot 1	32	25	3-*α*, *μ* 22-*α*, *κ*, *μ*, *ξ*	7	0	0	7	0	0	7	0	0
Spot 2	68	42	11-*α*, *μ* 2 31-*α*, *κ*, *μ*, *ξ*	26	0	0	22	0	0	22	0	0
Spot 3	24	13	1-*α*, *μ* 12-*α*, *κ*, *μ*, *ξ*	11	0	0	11	0	0	11	0	0
Spot 4	9	0	0	9	0	0	9	0	0	9	0	0
Total		80 (100%)			0 (0%)			0 (0%)			0 (0%)	

**Table 4:** Tropomyosin peptides identified in adult monkey heart #2.

Spot	TPM1			TPM2			TPM3			TPM4		
	Total	Unique	Isoform	Total	Unique	Isoform	Total	Unique	Isoform	Total	Unique	Isoform
Spot 1	14	4	4- *α*, *κ*, *μ*, *ξ*	6	0	0	2	0	0	2	0	0
Spot 2	73	40	3-*α*, *μ* 37-*α*, *κ*, *μ*, *ξ*	30	0	0	22	0	0	19	0	0
Spot 3	87	54	46- *α*, *κ*, *μ*, *ξ* 8-*α*, *μ*	32	0	0	25	0	0	25	0	0
Spot 4	100	60	14- *α*, *μ* 46-*α*, *κ*, *μ*, *ξ*	45	0	0	42	0	0	42	0	0
Total		158 (100%)			0 (0%)			0 (0%)			0 (0%)	

## Data Availability

The data generated and analyzed during the current study are available from the corresponding author upon request.

## Supplementary Materials

Supplementary Materials Amplification of various TPM1 isoforms by RT-PCR and/or nested RT-PCR with isoform specific primer-pair(s). (A) cDNAs made from total RNA of Cyn heart or skeletal muscle with oligo-dT were amplified with TPM1 exon 1A(+)/TPM1 exon 9B(−) primer pair that amplifies TPM1*α*, TPM1*κ*, TPM1*μ*, and TPM1*ξ*. (a) lane 1: heart; lane 2: skeletal muscle; lane 3: primer control. (B) Isolated DNA from lane 1 or lane 2 of Figure 2A was diluted and subsequently amplified with TPM1. Exon 2A(+)/Exon 9B(−) for TPM1*κ* or TPM1*ξ* (lanes 1 and 2 of Figure 2B, where lane 3 is primer control). Similarly, isolated and subsequently diluted DNA from lanes 1 or 2 of Figure 2A was amplified with TPM1. Exon 2B(+)/Exon 9B(−) for TPM1*α* or TPM1*μ* lane 4 and lane 5 of Figure 2B, where lane 6 is primer control. (a) lane 1: heart; lane 2: skeletal muscle; Lane 3: primer control; lane 4: heart; lane 5: skeletal muscle; lane 6: primer control. (C) Amplified DNA from each lane as shown in Figure 2A (lane 1 for heart and lane 2 for skeletal muscle) was gel extracted and further amplified with TPM1exon 1A(+)/exon 2A(−) primer pair for amplification of TPM1*κ* and TPM1*ξ*. (a) lane 1: heart; lane 2: skeletal muscle; lane 3: primer control. (D) Amplification of TPM1*κ* and TPM1*α* in Cyn heart and skeletal muscle. The initial amplified DNA as shown in Figure 2A was further amplified with TPM1exon 2A(+)/TPM1exon 3-4(−) for TPM1*κ* and/or TPM1*ξ* in heart (lane 1) and skeletal muscle (lane 2). The initial amplified DNA (as in Figure 2A) was amplified with TPM1exon 2B(+)/TPM1exon 3-4(−) for TPM1*α* or TPM1*μ* in heart (lane 4) and skeletal muscle (lane 5). TPM1*κ* or TPM1*ξ*: lane 1: heart, lane 2: skeletal muscle, and lane 3: primer control. TPM1*α* or TPM1*μ*: lane 4: heart, lane 5: skeletal muscle, and lane 6: primer control. (E) Amplification of TPM1*α*, TPM1*μ*, TPM1*κ*, and TPM1*ξ*. The initial amplified DNA (as in Figure 2A) was further amplified with TPM1exon 6A(+)/TPM1exon 9B (−) for TPM1*μ* or TPM1*ξ*. Absence of a visible band suggests the absence of TPM1*μ* or TPM1*ξ* in monkey heart (lane 1) and skeletal muscle (lane 2). The initial amplicon DNA was also further amplified with TPM1exon 6B(+)/TPM1exon 9B(−) that target TPM1*α* and TPM1*κ*. A strong band in lane 4 (heart) and lane 5 (skeletal muscle) suggests the presence of TPM1*α* and/or TPM1*κ*. TPM1*μ* and/or TPM1*ξ*: lane 1: heart, lane 2: skeletal muscle, and lane 3: primer control. TPM1*α* and/or TPM1*κ*: lane 4: heart, lane 5: skeletal muscle, and lane 6: primer control. *Supplementary Figure 2*. Expression of TPM3*α* and TPM3 in Cyn heart and skeletal muscle. (A). cDNAs were made with RNA from Cyn heart and skeletal muscle using oligo dT. First PCR amplification was carried out with TPM3 exon 1A(+)/TPM3 exon 9B(−) primer pair. Subsequently nested PCR was carried out with TPM3 exon1A(+)/TPM3 exon9B(−) primer-pair (Figure 5A). Lane 1: heart, lane 2: skeletal muscle, and lane 3: primer control. (B) The amplified DNA from Figure 5A was diluted and further amplified with TPM3 exon 6A(+)/TPM3 exon 9A(−) for TPM3] (Figure 5B) and by TPM3 exon 6B(+)/TPM3 exon 9B(−) primer-pair for TPM3*α*. Lane 1: heart, lane 2: skeletal muscle, and lane 3: primer control for TPM3n. Lane 4: heart, lane 5: skeletal muscle, and lane 6: primer control for TPM3*α*. The results in Figure 1A show that both heart (lane 1) and skeletal muscle (lane 2) express high molecular weight TPM1 transcripts which, after direct DNA sequence analyses, revealed the presence of TPM1a (Figure 1A) indicating that it may be the most dominant TPM1 isoform. The results in Figure 1B (lane 1 and lane 2) show that both heart and skeletal muscle of monkey express TPM1k. The results depicted in Figure 1C suggested a slightly higher expression of TPM1k in monkey heart compared to skeletal muscle. The results shown in Figure 1D also suggest that the expression of TPM1k is slightly higher in Cyn monkey heart compared to skeletal muscle. However, the level of expression of TPM1a in Cyn monkey heart and skeletal muscle is similar. The results depicted in Figure 1E show absence of high molecular weight TPM1 isoform with Exon 6A, as no band is visible in lane 1 (heart) and lane 2 (skeletal muscle). These results indicate a lack of expression of TPM1*μ* and TPM1x in Cyn monkey heart and skeletal muscles. On the contrary, a strong amplification in heart (lane 4) and skeletal muscle (lane 5) with primer pair TPM1exon 6B (+)/ TPM1exon 9A (-) indicates the expression of TPM1a or TPM1k in Cyn heart and skeletal muscle. In fact, the nucleotide sequence analyses (Figure 2A and 2B) uphold the RT- PCR data. Figure 2A depicts the amplified TPM3 DNA both in Cyn monkey heart (lane 1) and skeletal muscle (lane 2). The amplified DNA could be from TPM3a or TPM3n or both. The results presented in Figure 2B show that there is no visible amplicon of the correct size with TPM3 exon 6A (+) / TPM3 exon 9B (-) primer-pair. A lack of the PCR products with exon 6A primer suggest that there may not be detectable expression of TPM3n in monkey heart (lane 1, Figure 2B) and skeletal muscle (lane 2, Figure 2B). The results show the expression of TPM3a in Cyn heart and skeletal muscles. The expression level was much lower in Cyn monkey heart compared to skeletal muscle. The nucleotide sequence analyses confirmed Cyn monkey TPM3a expression. The nucleotide as well as deduced amino sequence are given in Figure 4. It is to be noted that the TPM3a amino acid sequence of Cyn and human are 100% identical. *Supplementary Figure 3*. Comparison of nucleotide sequences of Cyn TPM1*α*, TPM1*κ*, TPM2*α*, TPM3*α*, and TPM4*α* with the corresponding sequences of humans by best fit. (A) Cyn TPM1*α* vs. human TPM1*α*. (B) Cyn TPM1*κ* vs. human TPM1*κ*. (C) Cyn TPM2*α* vs. human TPM2*α*. (D) Cyn TPM3*α* vs. human TPM3*α*. (E) Cyn TPM4*α* vs. human TPM4*α*. *Supplementary Figure 4*. 2D Western blot analyses with extracts from adult Cyn heart. (A) The Coomassie stained monkey adult cardiac muscle protein across the gel. (B) The PVDF filter was stained with CH1 monoclonal antibody followed by treatment with a secondary antibody as stated under materials and methods, and subsequently treated with ECL and exposed to X-ray film. Developed X-ray film was superimposed on the top of the Coomassie stained second gel as well as on the Coomassie stained PVDF filter. Four spots, 1, 2, 3, and 4, were marked, excised, and used for extraction of protein for subsequent mass spectra analyses. *Supplementary Figure 5*. Identification of amino acid sequences from the peptides extracted from spots 1, 2, 3, and 4 after 2D western blot analyses of adult Cyn heart (#1) protein with CH1 monoclonal antibody. Red color letters indicate peptide sequences identified by mass spectra. Their location within the entire peptide sequence of TPM1*α* is shown. *Supplementary Figure 6*. 2D Western blot analyses with extracts from adult Cyn heart (#2). (A) The Coomassie stained monkey adult heart (#2) protein across gel. (B) The PVDF filter was stained with CH1 monoclonal antibody followed by treatment with secondary antibody as stated under materials and methods, and subsequently treated with ECL and exposed to X-ray film. Developed X-ray film was superimposed on the top of the Coomassie stained second gel as well as on the Coomassie stained PVDF filter. Four spots 1, 2, 3, and 4 were marked, excised, and used for extraction of protein for subsequent mass spectrometric analyses. *Supplementary Figure 7*. Identification of amino acid sequences from the peptides extracted from spots 1, 2, 3, and 4 after 2D Western blot analyses of adult Cyn heart (#2) protein with CH1 monoclonal antibody. Red color letters indicate peptide sequences identified by mass spectra. Their location within the entire peptide sequence of TPM1*α* is shown. *Supplementary Table 1*. Size of the PCR products amplified by various TPM1 and TPM3 primer pairs used in this study.
