# Supplementary material for: Tropomyosin Isoform Diversity in the Cynomolgus Monkey Heart and Skeletal Muscles Compared to Human Tissues
Source: Biochem Res Int. 2023 Jan 24;2023:1303500. doi: 10.1155/2023/1303500 (PMC9889151; doi:10.1155/2023/1303500)
Supplement: Supplementary Materials — Amplification of various TPM1 isoforms by RT-PCR and/or nested RT-PCR with isoform specific primer-pair(s). (A) cDNAs made from total RNA of Cyn heart or skeletal muscle with oligo-dT were amplified with TPM1 exon 1A(+)/TPM1 exon 9B(−) primer pair that amplifies TPM1α, TPM1κ, TPM1μ, and TPM1ξ. (a) lane 1: heart; lane 2: skeletal muscle; lane 3: primer control. (B) Isolated DNA from lane 1 or lane 2 of Figure 2A was diluted and subsequently amplified with TPM1. Exon 2A(+)/Exon 9B(−) for TPM1κ or TPM1ξ (lanes 1 and 2 of Figure 2B, where lane 3 is primer control). Similarly, isolated and subsequently diluted DNA from lanes 1 or 2 of Figure 2A was amplified with TPM1. Exon 2B(+)/Exon 9B(−) for TPM1α or TPM1μ lane 4 and lane 5 of Figure 2B, where lane 6 is primer control. (a) lane 1: heart; lane 2: skeletal muscle; Lane 3: primer control; lane 4: heart; lane 5: skeletal muscle; lane 6: primer control. (C) Amplified DNA from each lane as shown in Figure 2A (lane 1 for heart and lane 2 for skeletal muscle) was gel extracted and further amplified with TPM1exon 1A(+)/exon 2A(−) primer pair for amplification of TPM1κ and TPM1ξ. (a) lane 1: heart; lane 2: skeletal muscle; lane 3: primer control. (D) Amplification of TPM1κ and TPM1α in Cyn heart and skeletal muscle. The initial amplified DNA as shown in Figure 2A was further amplified with TPM1exon 2A(+)/TPM1exon 3-4(−) for TPM1κ and/or TPM1ξ in heart (lane 1) and skeletal muscle (lane 2). The initial amplified DNA (as in Figure 2A) was amplified with TPM1exon 2B(+)/TPM1exon 3-4(−) for TPM1α or TPM1μ in heart (lane 4) and skeletal muscle (lane 5). TPM1κ or TPM1ξ: lane 1: heart, lane 2: skeletal muscle, and lane 3: primer control. TPM1α or TPM1μ: lane 4: heart, lane 5: skeletal muscle, and lane 6: primer control. (E) Amplification of TPM1α, TPM1μ, TPM1κ, and TPM1ξ. The initial amplified DNA (as in Figure 2A) was further amplified with TPM1exon 6A(+)/TPM1exon 9B (−) for TPM1μ or TPM1ξ. Absence of a visible band suggests the absen [file 1303500.f1.zip › supplementary figures/Sup.Figure 3B.docx]

. . . . .

1 atggacgccatcaagaagaagatgcagatgctgaagctcgacaaggagaa 50 Human

||||||||||||||||||||||||||||||||||||||||||||||||||

1 ATGGACGCCATCAAGAAGAAGATGCAGATGCTGAAGCTCGACAAGGAGAA 50. Cyn

. . . . .

51 cgccttggatcgagctgagcaggcggaggccgacaagaaggcggcggaag 100

||||||||||||||||||||||||||||||||||||||||||||||||||

51 CGCCTTGGATCGAGCTGAGCAGGCGGAGGCCGACAAGAAGGCGGCGGAAG 100

. . . . .

101 acaggagcaagcagctcgaggaggacatcgcggccaaggagaagttgctg 150

||||||||||||||||||||||||||||||||||||||||||||||||||

101 ACAGGAGCAAGCAGCTCGAGGAGGACATCGCGGCCAAGGAGAAGTTGCTG 150

. . . . .

151 cgggtgtcggaggacgagcgggaccgggtgctggaggagctgcacaaggc 200

||||||||||||||||||||||||||||||||||||||||||||||||||

151 CGGGTGTCGGAGGACGAGCGGGACCGGGTGCTGGAGGAGCTGCACAAGGC 200

. . . . .

201 ggaggacagcctcctggc**c**gccga**a**gaggccgccgccaaggctgaagccg 250

|||||||||||||||||| ||||| |||||||||||||||||||||||||

201 GGAGGACAGCCTCCTGGC**T**GCCGA**G**GAGGCCGCCGCCAAGGCTGAAGCCG 250

. . . . .

251 a**c**gtagcttctctgaacagacgcatccagct**g**gttgaggaagagttggat 300

| ||||||||||||||||||||||||||||| ||||||||||||||||||

251 A**T**GTAGCTTCTCTGAACAGACGCATCCAGCT**C**GTTGAGGAAGAGTTGGAT 300

. . . . .

301 cgtgcccaggagcgtctggcaacagctttgcagaagctggaggaagctga 350

||||||||||||||||||||||||||||||||||||||||||||||||||

301 CGTGCCCAGGAGCGTCTGGCAACAGCTTTGCAGAAGCTGGAGGAAGCTGA 350

. . . . .

351 gaaggcagcagatgagagtgagagaggcatgaaagtcattgagagtcgag 400

||||||||||||||||||||||||||||||||||||||||||||||||||

351 GAAGGCAGCAGATGAGAGTGAGAGAGGCATGAAAGTCATTGAGAGTCGAG 400

. . . . .

401 cccaaaa**a**gatgaagaaaaaatggaaattcaggagatccaactgaaagag 450

||||||| ||||||||||||||||||||||||||||||||||||||||||

401 CCCAAAA**G**GATGAAGAAAAAATGGAAATTCAGGAGATCCAACTGAAAGAG 450

. . . . .

451 gc**c**aagcacattgctgaagatgccgaccgcaaata**c**gaagaggtggcccg 500

|| |||||||||||||||||||||||||||||||| ||||||||||||||

451 GC**T**AAGCACATTGCTGAAGATGCCGACCGCAAATA**T**GAAGAGGTGGCCCG 500

. . . . .

501 taagctggtcatcattgagag**c**gacctggaacgtgcagaggagcgggctg 550

||||||||||||||||||||| ||||||||||||||||||||||||||||

501 TAAGCTGGTCATCATTGAGAG**T**GACCTGGAACGTGCAGAGGAGCGGGCTG 550

. . . . .

551 agctctcagaaggcaaatgtgccgagcttgaagaagaattgaaaactgtg 600

||||||||||||||||||||||||||||||||||||||||||||||||||

551 AGCTCTCAGAAGGCAAATGTGCCGAGCTTGAAGAAGAATTGAAAACTGTG 600

. . . . .

601 acgaac**g**acttgaagtcactggaggctcaggctgagaagtactcgcagaa 650

|||||| |||||||||||||||||||||||||||||||||||||||||||

601 ACGAAC**A**ACTTGAAGTCACTGGAGGCTCAGGCTGAGAAGTACTCGCAGAA 650

. . . . .

651 ggaagac**a**gatatgaggaagagatcaaggtcctttccgacaagctgaagg 700

|||||||| |||||||||||||||||||||||||||||||||||||||||

651 GGAAGAC**A**AATATGAGGAAGAGATCAAGGTCCTTTCCGACAAGCTGAAGG 700

. . . . .

701 aggctgagactcgggctgagtttgcggagaggtcagtaactaaattggag 750

||||||||||||||||||||||||||||||||||||||||||||||||||

701 AGGCTGAGACTCGGGCTGAGTTTGCGGAGAGGTCAGTAACTAAATTGGAG 750

. . . . .

751 aaaagcattgatgacttagaagacgagctgtacgctcagaaactgaagta 800

||||||||||||||||||||||||||||||||||||||||||||||||||

751 AAAAGCATTGATGACTTAGAAGACGAGCTGTACGCTCAGAAACTGAAGTA 800

. . . . .

801 caaagccatcagcgaggagctggaccacgctctcaacgatatgacttcca 850

||||||||||||||||||||||||||||||||||||||||||||||||||

801 CAAAGCCATCAGCGAGGAGCTGGACCACGCTCTCAACGATATGACTTCCA 850

851 tataa 855

|||||

851 TATAA 855

**Sup.Figure 3B. Comparison of nucleotide sequences of TPM1κ with the corresponding sequences of humans by best fit.**
