# Supplementary material for: Tropomyosin Isoform Diversity in the Cynomolgus Monkey Heart and Skeletal Muscles Compared to Human Tissues
Source: Biochem Res Int. 2023 Jan 24;2023:1303500. doi: 10.1155/2023/1303500 (PMC9889151; doi:10.1155/2023/1303500)
Supplement: Supplementary Materials — Amplification of various TPM1 isoforms by RT-PCR and/or nested RT-PCR with isoform specific primer-pair(s). (A) cDNAs made from total RNA of Cyn heart or skeletal muscle with oligo-dT were amplified with TPM1 exon 1A(+)/TPM1 exon 9B(−) primer pair that amplifies TPM1α, TPM1κ, TPM1μ, and TPM1ξ. (a) lane 1: heart; lane 2: skeletal muscle; lane 3: primer control. (B) Isolated DNA from lane 1 or lane 2 of Figure 2A was diluted and subsequently amplified with TPM1. Exon 2A(+)/Exon 9B(−) for TPM1κ or TPM1ξ (lanes 1 and 2 of Figure 2B, where lane 3 is primer control). Similarly, isolated and subsequently diluted DNA from lanes 1 or 2 of Figure 2A was amplified with TPM1. Exon 2B(+)/Exon 9B(−) for TPM1α or TPM1μ lane 4 and lane 5 of Figure 2B, where lane 6 is primer control. (a) lane 1: heart; lane 2: skeletal muscle; Lane 3: primer control; lane 4: heart; lane 5: skeletal muscle; lane 6: primer control. (C) Amplified DNA from each lane as shown in Figure 2A (lane 1 for heart and lane 2 for skeletal muscle) was gel extracted and further amplified with TPM1exon 1A(+)/exon 2A(−) primer pair for amplification of TPM1κ and TPM1ξ. (a) lane 1: heart; lane 2: skeletal muscle; lane 3: primer control. (D) Amplification of TPM1κ and TPM1α in Cyn heart and skeletal muscle. The initial amplified DNA as shown in Figure 2A was further amplified with TPM1exon 2A(+)/TPM1exon 3-4(−) for TPM1κ and/or TPM1ξ in heart (lane 1) and skeletal muscle (lane 2). The initial amplified DNA (as in Figure 2A) was amplified with TPM1exon 2B(+)/TPM1exon 3-4(−) for TPM1α or TPM1μ in heart (lane 4) and skeletal muscle (lane 5). TPM1κ or TPM1ξ: lane 1: heart, lane 2: skeletal muscle, and lane 3: primer control. TPM1α or TPM1μ: lane 4: heart, lane 5: skeletal muscle, and lane 6: primer control. (E) Amplification of TPM1α, TPM1μ, TPM1κ, and TPM1ξ. The initial amplified DNA (as in Figure 2A) was further amplified with TPM1exon 6A(+)/TPM1exon 9B (−) for TPM1μ or TPM1ξ. Absence of a visible band suggests the absen [file 1303500.f1.zip › supplementary figures/Sup.Figure 3C.docx]

1 atggacgccatcaagaagaagatgcagatgctgaagctggacaaggagaa 50. Hunan TPM2α

||||||||||||||||||||||||||||||||||||||||||||||||||

1 atggacgccatcaagaagaagatgcagatgctgaagctggacaaggagaa 50. Cyn TPM2α

. . . . .

51 cgccatcgaccgcgccgagcaggccgaagccgacaagaagcaagctgagg 100

||||||||||||||||||||||||||||||||||||||||||||||||||

51 cgccatcgaccgcgccgagcaggccgaagccgacaagaagcaagctgagg 100

. . . . .

101 a**c**cgctgcaagcagctggaggaggagcagcaggccctccagaagaagctg 150

| ||||||||||||||||||||||||||||||||||||||||||||||||

101 a**t**cgctgcaagcagctggaggaggagcagcaggccctccagaagaagctg 150

. . . . .

151 aaggggacagaggatga**g**gtggaaaagtattctgaatc**c**gtgaaggaggc 200

||||||||||||||||| |||||||||||||||||||| |||||||||||

151 aaggggacagaggatga**a**gtggaaaagtattctgaatc**t**gtgaaggaggc 200

. . . . .

201 ccaggagaaactggagcaggccgagaagaaggccac**t**gatgctgaggcag 250

|||||||||||||||||||||||||||||||||||| |||||||||||||

201 ccaggagaaactggagcaggccgagaagaaggccac**c**gatgctgaggcag 250

. . . . .

251 atgtggcctccctgaaccgccgcattcagctggt**t**gaggaggagctggac 300

|||||||||||||||||||||||||||||||||| |||||||||||||||

251 atgtggcctccctgaaccgccgcattcagctggt**a**gaggaggagctggac 300

. . . . .

301 cgggcccaggagcgcctggctacagccctgcagaagctggaggaggccga 350

||||||||||||||||||||||||||||||||||||||||||||||| ||

301 cgggcccaggagcgcctggctacagccctgcagaagctggaggaggc**t**ga 350

. . . . .

351 gaaggc**g**gctgatgagagcgagagaggaatgaaggtcatcgaaaaccggg 400

|||||| |||||||||||||||||||||||||||||||||||||||||||

351 gaaggc**a**gctgatgagagcgagagaggaatgaaggtcatcgaaaaccggg 400

. . . . .

451 ccatgaagga**t**gaggagaagatgga**a**ctgcaggagatgcagctgaa**g**gag 450

|||||||||| |||||||||||||| |||||||||||||||||||| |||

401 ccatgaagga**c**gaggagaagatggagctgcaggagatgcagctgaa**a**gag 450

. . . . .

451 gccaagcacatcgctgaggattcagaccgcaaatatga**a**gaggtggccag 500

|||||||||||||||||||||||||||||||||||||| |||||||||||

451 gccaagcacatcgctgaggattcagaccgcaaatatga**g**gaggtggccag 500

. . . . .

501 gaagctggt**g**atcctggaaggagagctggagcgctc**g**ga**g**gagag**g**gctg 550

||||||||| |||||||||||||||||||||||||| || ||||| ||||

501 gaagctggt**t**atcctggaaggagagctggagcgctcagaagagag**a**gctg 550

. . . . .

551 aggtggccgagagtaaatgtggggacctagaggaggagctgaaaattgtt 600

||||||||||||||||||||||||||||||||||||||||||||||||||

551 aggtggccgagagtaaatgtggggacctagaggaggagctgaaaattgtt 600

. . . . .

601 accaacaacttgaaatccctggaggcccaggcggacaagtattccaccaa 650

||||||||||||||||||||||||||||||||||||||||||||||||||

601 accaacaacttgaaatccctggaggcccaggcggacaagtattccaccaa 650

. . . . .

651 agaagataaatatgaagaggagatcaaactg**t**tggaggagaagctgaa**g**g 700

||||||||||||||||||||||||||||||| |||||||||||||||| |

651 agaagataaatatgaagaggagatcaaactg**c**tggaggagaagctgaa**a**g 700

. . . . .

701 aggctgagac**c**cgagcagagtttgccga**g**aggtctgtggcaaagttggag 750

|||||||||| ||||||||||||||||| |||||||||||||||||||||

701 aggctgagac**t**cgagcagagtttgccga**a**aggtctgtggcaaagttggag 750

. . . . .

751 aaaaccatcgatgacct**a**gaaga**t**gaagtctatgcccagaagatgaagta 800

||||||||||||||||| ||||| ||||||||||||||||||||||||||

751 aaaaccatcgatgacct**g**gaaga**c**gaagtctatgcccagaagatgaagta 800

. . . . .

801 caaggccattagcgagga**a**ctggacaacgcactcaatgacatcacctccc 850

|||||||||||||||||| |||||||||||||||||||||||||||||||

801 caaggccattagcgagga**g**ctggacaacgcactcaatgacatcacctccc 850

1090 tctga 1094

|||||

851 tctga 855

**Sup.Figure 3C. Comparison of nucleotide sequences of TPM2α with the corresponding sequences of humans by best fit.**
