# Supplementary material for: Tropomyosin Isoform Diversity in the Cynomolgus Monkey Heart and Skeletal Muscles Compared to Human Tissues
Source: Biochem Res Int. 2023 Jan 24;2023:1303500. doi: 10.1155/2023/1303500 (PMC9889151; doi:10.1155/2023/1303500)
Supplement: Supplementary Materials — Amplification of various TPM1 isoforms by RT-PCR and/or nested RT-PCR with isoform specific primer-pair(s). (A) cDNAs made from total RNA of Cyn heart or skeletal muscle with oligo-dT were amplified with TPM1 exon 1A(+)/TPM1 exon 9B(−) primer pair that amplifies TPM1α, TPM1κ, TPM1μ, and TPM1ξ. (a) lane 1: heart; lane 2: skeletal muscle; lane 3: primer control. (B) Isolated DNA from lane 1 or lane 2 of Figure 2A was diluted and subsequently amplified with TPM1. Exon 2A(+)/Exon 9B(−) for TPM1κ or TPM1ξ (lanes 1 and 2 of Figure 2B, where lane 3 is primer control). Similarly, isolated and subsequently diluted DNA from lanes 1 or 2 of Figure 2A was amplified with TPM1. Exon 2B(+)/Exon 9B(−) for TPM1α or TPM1μ lane 4 and lane 5 of Figure 2B, where lane 6 is primer control. (a) lane 1: heart; lane 2: skeletal muscle; Lane 3: primer control; lane 4: heart; lane 5: skeletal muscle; lane 6: primer control. (C) Amplified DNA from each lane as shown in Figure 2A (lane 1 for heart and lane 2 for skeletal muscle) was gel extracted and further amplified with TPM1exon 1A(+)/exon 2A(−) primer pair for amplification of TPM1κ and TPM1ξ. (a) lane 1: heart; lane 2: skeletal muscle; lane 3: primer control. (D) Amplification of TPM1κ and TPM1α in Cyn heart and skeletal muscle. The initial amplified DNA as shown in Figure 2A was further amplified with TPM1exon 2A(+)/TPM1exon 3-4(−) for TPM1κ and/or TPM1ξ in heart (lane 1) and skeletal muscle (lane 2). The initial amplified DNA (as in Figure 2A) was amplified with TPM1exon 2B(+)/TPM1exon 3-4(−) for TPM1α or TPM1μ in heart (lane 4) and skeletal muscle (lane 5). TPM1κ or TPM1ξ: lane 1: heart, lane 2: skeletal muscle, and lane 3: primer control. TPM1α or TPM1μ: lane 4: heart, lane 5: skeletal muscle, and lane 6: primer control. (E) Amplification of TPM1α, TPM1μ, TPM1κ, and TPM1ξ. The initial amplified DNA (as in Figure 2A) was further amplified with TPM1exon 6A(+)/TPM1exon 9B (−) for TPM1μ or TPM1ξ. Absence of a visible band suggests the absen [file 1303500.f1.zip › supplementary figures/Sup.Figure 3D (2).docx]

. . . . .

1 ATGATGGAGGCCATCAAGAAAAAGATGCAGATGCTGAAGTTAGA**C**AAGGA 50. Human TPM3α

|||||||||||||||||||||||||||||||||||||||||||| |||||

1 ATGATGGAGGCCATCAAGAAAAAGATGCAGATGCTGAAGTTAGA**T**AAGGA 50. Cyn TPM3α

. . . . .

51 GAATGCTCTGGATCGGGCAGAGCAAGCTGAAGCTGAGCAGAAGCAGGCAG 100

||||||||||||||||||||||||||||||||||||||||||||||||||

51 GAATGCTCTGGATCGGGCAGAGCAAGCTGAAGCTGAGCAGAAGCAGGCAG 100

. . . . .

101 AAGAAAGAAGTAAACAGCTGGAGGATGAGCTGGCAGCCATGCAGAAGAAG 150

|||||||||||||||||||||||||||||||||||||||||||||||||

101 AAGAAAGAAGTAAACAGCTGGAGGATGAGCTGGCAGCCATGCAGAAGAAA 150

. . . . .

151 CTGAAAGGGACAGAGGATGAGCTGGACAAGTATTC**T**GAAGCTTTGAAGGA 200

||||||||||||||||||||||||||||||||||| ||||||||||||||

151 CTGAAAGGGACAGAGGATGAGCTGGACAAGTATTC**A**GAAGCTTTGAAGGA 200

. . . . .

201 TGCCCAGGAGAAGCTGGAACTGGCAGAGAAGAAGGCTGCTGATGCTGAGG 250

||||||||||||||||||||||||||||||||||||||||||||||||||

201 TGCCCAGGAGAAGCTGGAACTGGCAGAGAAGAAGGCTGCTGATGCTGAGG 250

. . . . .

251 CTGAGGTGGCCTCCTTGAACCGTAGGATCCAGCTGGTTGAAGAAGAGATG 300

||||||||||||||||||||||||||||||||||||||||||| ||| ||

251 CTGAGGTGGCCTCCTTGAACCGTAGGATCCAGCTGGTTGAAGAGGAGCTG 300

. . . . .

301 GACCGTGCTCAGGAGCG**C**CTGGCCACTGCCCTGCAAAA**G**CTGGAAGAAGC 350

||||||||||||||||| |||||||||||||||||||| |||||||||||

301 GACCGTGCTCAGGAGCG**G**CTGGCCACTGCCCTGCAAAA**A**CTGGAAGAAGC 350

. . . . .

351 TGAAAAAGCTGCTGATGAGAGTGAGAGAGGTATGAAGGTTATTGAAAACC 400

|||||||||||||||||||||||||||||||||||||||||||||||||

351 AGAAAAAGCTGCTGATGAGAGTGAGAGAGGTATGAAGGTTATTGAAAACC 400

. . . . .

401 GGGCCTTAAAAGATGAAGAAAAGATGGAACTCCAGGAAATCCAACTCAAA 450

||||||||||||||||||||||||||||||||||||||||||||||||||

401 GGGCCTTAAAAGATGAAGAAAAGATGGAACTCCAGGAAATCCAACTCAAA 450

. . . . .

451 GAAGCTAAGCACATTGCAGAAGAGGCAGATAGGAAGTATGAAGAGGTGGC 500

||||||||||||||||||||||||||||||||||||||||||||||||||

451 GAAGCTAAGCACATTGCAGAAGAGGCAGATAGGAAGTATGAAGAGGTGGC 500

. . . . .

501 TCGTAA**G**TTGGTGATCATTGAAGGAGACTTGGAACGCACAGAGGAACGAG 550

|||||| |||||||||||||||||||||||||||||||||||||||||||

501 TCGTAA**A**TTGGTGATCATTGAAGGAGACTTGGAACGCACAGAGGAACGAG 550

. . . . .

551 CTGAGCTGGCAGAGTCTAAGTGTTCTGAGCTGGAGGAGGAGCTGAAGAAT 600

||||||||||||||||||||||||||||||||||||||||||||||||||

551 CTGAGCTGGCAGAGTCTAAGTGTTCTGAGCTGGAGGAGGAGCTGAAGAAT 600

. . . . .

601 GTCACCAACAACCTCAAGTCTCTTGAGGCTCAGGCGGAGAAGTACTCTCA 650

||||||||||||||||||||||||||||||||||||||||||||||||||

601 GTCACCAACAACCTCAAGTCTCTTGAGGCTCAGGCGGAGAAGTACTCTCA 650

. . . . .

651 AAAAGAAGA**T**AAATATGAGGAAGA**A**ATCAAGATTCTTACTGATAAACTCA 700

||||||||| |||||||||||||| |||||||||||||||||||||||||

651 AAAAGAAGA**C**AAATATGAGGAAGA**G**ATCAAGATTCTTACTGATAAACTCA 700

. . . . .

701 AGGAGGCAGAGACCCGTGCTGAGTTTGCTGAGAGATCGGTAGCCAAGCTG 750

||||||||||||||||||||||||||||||||||||||||||||||||||

701 AGGAGGCAGAGACCCGTGCTGAGTTTGCTGAGAGATCGGTAGCCAAGCTG 750

. . . . .

751 GAAAAGACAATTGATGAC**C**TGGAAGATGAGCTCTATGCCCAGAAACTGAA 800

|||||||||||||||||| |||||||||||||||||||||||||||||||

751 GAAAAGACAATTGATGAC**T**TGGAAGATGAGCTCTATGCCCAGAAACTGAA 800

. . . . .

801 GTACAAGGCCATTAGCGAGGA**G**CTGGACCACGCCCTCAATGACATGACCT 850

||||||||||||||||||||| ||||||||||||||||||||||||||||

801 GTACAAGGCCATTAGCGAGGAACTGGACCACGCCCTCAATGACATGACCT 850

851 CTATAT 856

||||||

851 CTATAT 856

**Sup.Figure 3D. Comparison of nucleotide sequences of TPM3α with the corresponding sequences of humans by best fit.**
