# Supplementary material for: Tropomyosin Isoform Diversity in the Cynomolgus Monkey Heart and Skeletal Muscles Compared to Human Tissues
Source: Biochem Res Int. 2023 Jan 24;2023:1303500. doi: 10.1155/2023/1303500 (PMC9889151; doi:10.1155/2023/1303500)
Supplement: Supplementary Materials — Amplification of various TPM1 isoforms by RT-PCR and/or nested RT-PCR with isoform specific primer-pair(s). (A) cDNAs made from total RNA of Cyn heart or skeletal muscle with oligo-dT were amplified with TPM1 exon 1A(+)/TPM1 exon 9B(−) primer pair that amplifies TPM1α, TPM1κ, TPM1μ, and TPM1ξ. (a) lane 1: heart; lane 2: skeletal muscle; lane 3: primer control. (B) Isolated DNA from lane 1 or lane 2 of Figure 2A was diluted and subsequently amplified with TPM1. Exon 2A(+)/Exon 9B(−) for TPM1κ or TPM1ξ (lanes 1 and 2 of Figure 2B, where lane 3 is primer control). Similarly, isolated and subsequently diluted DNA from lanes 1 or 2 of Figure 2A was amplified with TPM1. Exon 2B(+)/Exon 9B(−) for TPM1α or TPM1μ lane 4 and lane 5 of Figure 2B, where lane 6 is primer control. (a) lane 1: heart; lane 2: skeletal muscle; Lane 3: primer control; lane 4: heart; lane 5: skeletal muscle; lane 6: primer control. (C) Amplified DNA from each lane as shown in Figure 2A (lane 1 for heart and lane 2 for skeletal muscle) was gel extracted and further amplified with TPM1exon 1A(+)/exon 2A(−) primer pair for amplification of TPM1κ and TPM1ξ. (a) lane 1: heart; lane 2: skeletal muscle; lane 3: primer control. (D) Amplification of TPM1κ and TPM1α in Cyn heart and skeletal muscle. The initial amplified DNA as shown in Figure 2A was further amplified with TPM1exon 2A(+)/TPM1exon 3-4(−) for TPM1κ and/or TPM1ξ in heart (lane 1) and skeletal muscle (lane 2). The initial amplified DNA (as in Figure 2A) was amplified with TPM1exon 2B(+)/TPM1exon 3-4(−) for TPM1α or TPM1μ in heart (lane 4) and skeletal muscle (lane 5). TPM1κ or TPM1ξ: lane 1: heart, lane 2: skeletal muscle, and lane 3: primer control. TPM1α or TPM1μ: lane 4: heart, lane 5: skeletal muscle, and lane 6: primer control. (E) Amplification of TPM1α, TPM1μ, TPM1κ, and TPM1ξ. The initial amplified DNA (as in Figure 2A) was further amplified with TPM1exon 6A(+)/TPM1exon 9B (−) for TPM1μ or TPM1ξ. Absence of a visible band suggests the absen [file 1303500.f1.zip › supplementary figures/Sup.Figure 3E (2).docx]

. . . . .

1 atggaggccatcaagaagaaaatgcagatgctgaagttggacaaggagaa 50 Human TPM4α

||||||||||||||||||||||||||||||||||||||||||||||||||

1 atggaggccatcaagaagaaaatgcagatgctgaagttggacaaggagaa 50 Cyn TPM4α

. . . . .

51 tgccatcgaccgcgcggagcaggc**g**gaggcggataagaaagccgc**t**gagg 100

|||||||||||||||||||||||| |||||||||||||||||||| ||||

51 tgccatcgaccgcgcggagcaggc**c**gaggcggataagaaagccgc**c**gagg 100

. . . . .

101 acaagtgcaagcaggtggaggagga*g*ctgac*g*cacctccagaagaaacta 150

||||||||||||||||||||||||| ||||| ||||||||||||||||||

101 acaagtgcaagcaggtggaggagga*a*ctgac*a*cacctccagaagaaacta 150

. . . . .

151 aaagggacagaggacgagctggataaatattc**c**gaggac**c**tgaaggacgc 200

|||||||||||||||||||||||||||||||| ||| || ||||||||||

151 aaagggacagaggacgagctggataaatattc**g**gagaac**t**tgaaggacgc 200

. . . . .

201 gcaggagaag**c**tggagctcacggagaagaa**g**gcctccgacgctgaaggtg 250

|||||||||| ||||||||||||||||||| |||||||||||||||||||

201 gcaggagaag**t**tggagctcacggagaagaa**a**gcctccgacgctgaaggtg 250

. . . . .

251 atgtggccgccctcaaccgacgcatccagctcgttgaggaggagttggac 300

||||||||||||||||||||||||||||||||||||||||||||||||||

251 atgtggccgccctcaaccgacgcatccagctcgttgaggaggagttggac 300

. . . . .

301 agggctcaggaacgactggccacggccctgcagaagctggaggaggc**a**ga 350

||||||||||||||||||||||||||||||||||||||||||||||| ||

301 agggctcaggaacgactggccacggccctgcagaagctggaggaggc**g**ga 350

. . . . .

351 aaa**a**gctgcagatgagagtgagagaggaatgaaggtgatagaaaaccggg 400

||| ||||||||||||||||||||||||||||||||||||||||||||||

351 aaa**g**gctgcagatgagagtgagagaggaatgaaggtgatagaaaaccggg 400

. . . . .

401 ccatgaaggatgaggagaagatgga**g**attcaggagatgcagctcaaagag 450

||||||||||||||||||||||||| ||||||||||||||||||||||||

401 ccatgaaggatgaggagaagatgga**a**attcaggagatgcagctcaaagag 450

. . . . .

451 gccaagcacattgc**g**gaagaggctgaccgcaaatacgaggaggtagctcg 500

|||||||||||||| |||||||||||||||||||||||||||||||||||

451 gccaagcacattgc**a**gaagaggctgaccgcaaatacgaggaggtagctcg 500

. . . . .

501 taagctggtcatcctggagggtgagctggagagggcagaggagcgtgcgg 550

||||||||||||||||||||||||||||||||||||||||||||||||||

501 taagctggtcatcctggagggtgagctggagagggcagaggagcgtgcgg 550

. . . . .

551 aggtgtctgaactaaaatgtggtgacctggaagaagaactcaagaatgtt 600

||||||||||||||||||||||||||||||||||||||||||||||||||

551 aggtgtctgaactaaaatgtggtgacctggaagaagaactcaagaatgtt 600

. . . . .

601 actaacaa**t**ctgaaatctctggaggctgc**a**tctgaaaagtattctgaaaa 650

|||||||| |||||||||||||||||||| ||||||||||||||||||||

601 actaacaa**c**ctgaaatctctggaggctgc**g**tctgaaaagtattctgaaaa 650

. . . . .

651 ggaggacaaatatgaagaagaaattaaacttctgtctgacaaactgaaag 700

||||||||||||||||||||||||||||||||||||||||||||||||||

651 ggaggacaaatatgaagaagaaattaaacttctgtctgacaaactgaaag 700

. . . . .

701 aggctgagacccgtgctgaatttgcagagagaacggttgcaaaactggaa 750

||||||||||||||||||||||||||||||||||||||||||||||||||

701 aggctgagacccgtgctgaatttgcagagagaacggttgcaaaactggaa 750

. . . . .

751 aagacaattgatgacctggaagatgagttatacgctcagaagctcaagta 800

||||||||||||||||||||||||||||||||||||||||||||||||||

751 aagacaattgatgacctggaagatgagttatacgctcagaagctcaagta 800

. . . . .

801 caaagctatcagcgaggaactggaccac**g**ctctcaacgacatgacctctc 850

|||||||||||||||||||||||||||| |||||||||||||||||||||

801 caaagctatcagcgaggaactggaccac**a**ctctcaacgacatgacctctc 850

851 tctga 855

|||||

851 tctga 855

**Sup.Figure 3E. Comparison of nucleotide sequences of TPM4α with the corresponding sequences of humans by best fit.**
