# Supplementary material for: Tropomyosin Isoform Diversity in the Cynomolgus Monkey Heart and Skeletal Muscles Compared to Human Tissues
Source: Biochem Res Int. 2023 Jan 24;2023:1303500. doi: 10.1155/2023/1303500 (PMC9889151; doi:10.1155/2023/1303500)
Supplement: Supplementary Materials — Amplification of various TPM1 isoforms by RT-PCR and/or nested RT-PCR with isoform specific primer-pair(s). (A) cDNAs made from total RNA of Cyn heart or skeletal muscle with oligo-dT were amplified with TPM1 exon 1A(+)/TPM1 exon 9B(−) primer pair that amplifies TPM1α, TPM1κ, TPM1μ, and TPM1ξ. (a) lane 1: heart; lane 2: skeletal muscle; lane 3: primer control. (B) Isolated DNA from lane 1 or lane 2 of Figure 2A was diluted and subsequently amplified with TPM1. Exon 2A(+)/Exon 9B(−) for TPM1κ or TPM1ξ (lanes 1 and 2 of Figure 2B, where lane 3 is primer control). Similarly, isolated and subsequently diluted DNA from lanes 1 or 2 of Figure 2A was amplified with TPM1. Exon 2B(+)/Exon 9B(−) for TPM1α or TPM1μ lane 4 and lane 5 of Figure 2B, where lane 6 is primer control. (a) lane 1: heart; lane 2: skeletal muscle; Lane 3: primer control; lane 4: heart; lane 5: skeletal muscle; lane 6: primer control. (C) Amplified DNA from each lane as shown in Figure 2A (lane 1 for heart and lane 2 for skeletal muscle) was gel extracted and further amplified with TPM1exon 1A(+)/exon 2A(−) primer pair for amplification of TPM1κ and TPM1ξ. (a) lane 1: heart; lane 2: skeletal muscle; lane 3: primer control. (D) Amplification of TPM1κ and TPM1α in Cyn heart and skeletal muscle. The initial amplified DNA as shown in Figure 2A was further amplified with TPM1exon 2A(+)/TPM1exon 3-4(−) for TPM1κ and/or TPM1ξ in heart (lane 1) and skeletal muscle (lane 2). The initial amplified DNA (as in Figure 2A) was amplified with TPM1exon 2B(+)/TPM1exon 3-4(−) for TPM1α or TPM1μ in heart (lane 4) and skeletal muscle (lane 5). TPM1κ or TPM1ξ: lane 1: heart, lane 2: skeletal muscle, and lane 3: primer control. TPM1α or TPM1μ: lane 4: heart, lane 5: skeletal muscle, and lane 6: primer control. (E) Amplification of TPM1α, TPM1μ, TPM1κ, and TPM1ξ. The initial amplified DNA (as in Figure 2A) was further amplified with TPM1exon 6A(+)/TPM1exon 9B (−) for TPM1μ or TPM1ξ. Absence of a visible band suggests the absen [file 1303500.f1.zip › supplementary figures/Sup.Figure 7.docx]

Spot #1:16.5% TPM1a sequences are covered in Red

1 mdaikkkmqmlkldkenaldraeqaeadkkaaedrskqledelvslqkklkgtedeldky

61 sealkdaqeklelaekkatdaeadvaslnrriqlveeeldraqerlatalqkleeaekaa

121 desergmkviesraqkdeekmeiqeiqlkeakhiaedadrkyeevarklviiesdlerae

181 eraelsegkcaeleeelktvtnnlksleaqaekysqkedryeeeikvlsdklkeaetrae

241 faersvtkleksiddledelyaqklkykaiseeldhalndmtsi

Spot #2:65.14% TPM1a sequences are covered in red

1 mdaikkkmqmlkldkenaldraeqaeadkkaaedrskqledelvslqkklkgtedeldky

61 sealkdaqeklelaekkatdaeadvaslnrriqlveeeldraqerlatalqkleeaekaa

121 desergmkviesraqkdeekmeiqeiqlkeakhiaedadrkyeevarklviiesdlerae

181 eraelsegkcaeleeelktvtnnlksleaqaekysqkedryeeeikvlsdklkeaetrae

241 faersvtkleksiddledelyaqklkykaiseeldhalndmtsi

Spot #3:70.4% TPM1a sequences are covered in red

1 mdaikkkmqmlkldkenaldraeqaeadkkaaedrskqledelvslqkklkgtedeldky

61 sealkdaqeklelaekkatdaeadvaslnrriqlveeeldraqerlatalqkleeaekaa

121 desergmkviesraqkdeekmeiqeiqlkeakhiaedadrkyeevarklviiesdlerae

181 eraelsegkcaeleeelktvtnnlksleaqaekysqkedryeeeikvlsdklkeaetrae

241 faersvtkleksiddledelyaqklkykaiseeldhalndmtsi

Spot #4:87.67% TPM1a sequences are covered in red

1 mdaikkkmqmlkldkenaldraeqaeadkkaaedrskqledelvslqkklkgtedeldky

61 sealkdaqeklelaekkatdaeadvaslnrriqlveeeldraqerlatalqkleeaekaa

121 desergmkviesraqkdeekmeiqeiqlkeakhiaedadrkyeevarklviiesdlerae

181 eraelsegkcaeleeelktvtnnlksleaqaekysqkedryeeeikvlsdklkeaetrae

241 faersvtkleksiddledelyaqklkykaiseeldhalndmtsi

**Sup.Figure 7. Identification of amino acid sequences from the peptides extracted from Spots 1, 2, 3, and 4 after 2D western blot analyses of adult Cyn heart (#2) protein with CH1 monoclonal antibody.**
